# Serum amyloid P component promotes formation of distinct aggregated lysozyme morphologies and reduces toxicity in *Drosophila* flies expressing F57I lysozyme

**DOI:** 10.1371/journal.pone.0227227

**Published:** 2020-01-24

**Authors:** Liza Bergkvist, Daniel R. Richards, Ana Bernardo-Gancedo, Janet R. Kumita, Peter R. Nilsson, Ann-Christin Brorsson

**Affiliations:** 1 Division of Molecular Biotechnology, Department of Physics, Chemistry and Biology, Linköping University, Linköping, Sweden; 2 Centre for Misfolding Diseases, Department of Chemistry, University of Cambridge, Cambridge, England, United Kingdom; Consejo Superior de Investigaciones Cientificas, SPAIN

## Abstract

Many conflicting reports about the involvement of serum amyloid P component (SAP) in amyloid diseases have been presented over the years; SAP is known to be a universal component of amyloid aggregates but it has been suggested that it can both induce and suppress amyloid formation. By using our *Drosophila* model of systemic lysozyme amyloidosis, SAP has previously been shown to reduce the toxicity induced by the expression of the disease-associated lysozyme variant, F57I, in the *Drosophila* central nervous system. This study further investigates the involvement of SAP in modulating lysozyme toxicity using histochemistry and spectral analyses on the double transgenic WT and F57I lysozyme flies to probe; i) formation of aggregates, ii) morphological differences of the aggregated lysozyme species formed in the presence or absence of SAP, iii) location of lysozyme and iv) co-localisation of lysozyme and SAP in the fly brain. We found that SAP can counteract the toxicity (measured by the reduction in the median survival time) induced by F57I lysozyme by converting toxic F57I species into less toxic amyloid-like structures, as reflected by the spectral changes that p-FTAA undergoes when bound to lysozyme deposits in F57I-F57I-SAP flies as compared to F57I-F57I flies. Indeed, when SAP was introduced to *in vitro* lysozyme fibril formation, the endpoint fibrils had enhanced ThT fluorescence intensity as compared to lysozyme fibrils alone. This suggests that a general mechanism for SAP's role in amyloid diseases may be to promote the formation of stable, amyloid-like fibrils, thus decreasing the impact of toxic species formed along the aggregation pathway.

## Introduction

The serum amyloid P component (SAP) is known to be a universal component of amyloid plaques; however, conflicting data explaining its role in amyloid diseases have been reported. It has been proposed that SAP prevents proteolytic cleavage by binding to and stabilising aggregates [1]. It has also been suggested that SAP binds to misfolded species and prevents them from seeding larger aggregates [2], and that by binding to amyloid fibrils and their pre-aggregated precursors, SAP provides a defence mechanism against the formation of toxic species [3]. SAP has been closely linked to systemic amyloid diseases; in fact, radiolabelled SAP is used to monitor amyloid load in patients [4,5]. In a recent study, SAP was targeted in a treatment strategy for patients suffering from different systemic amyloid diseases, e.g. AA and AL amyloidosis; firstly, depletion of SAP circulating in the plasma was achieved by injecting the patients with the organic molecule CPHPC, then an anti-SAP antibody was introduced to target the remaining SAP within the amyloid deposits to trigger clearance of the amyloid load within the treated patients [6]. Taken together, the role of SAP in amyloid diseases still needs further investigation.

In the present study, a *Drosophila melanogaster* model of lysozyme amyloidosis was used to study the impact of co-expressing a disease-associated variant of lysozyme with SAP *in vivo*. Lysozyme amyloidosis is a hereditary systemic amyloid disease in which the bacteriolytic enzyme, lysozyme, misfolds due to mutations in its amino acid sequence resulting in the aggregation and deposition of up to kilograms of amyloid deposits. These amyloid aggregates frequently accumulate around the kidneys and liver of patients suffering from the disease, eventually leading to organ failure [7]. Lysozyme is a highly-expressed protein which can be found in bodily fluids such as tears, saliva and breast milk, where it plays an important part of the innate immune system [8]. There are eight disease-associated variants of lysozyme (Y54N, I56T, F57I, W64R, D67H, L84S (pL102S), T70N/F57I and T70N/W112R) which show increased tendencies to aggregate compared to the wild type (WT) protein [9]. The only treatment available for those suffering from the disease is organ transplantation; however, this does not cure the disease as the amyloid aggregates still continue to build up throughout the remainder of the patient's life [10]. *In vitro* studies of disease-associated lysozyme variants suggest that reductions in both the stability of the native state and in global co-operativity leads to the formation of transient, partially unfolded species that can aggregate and form amyloid fibrils [11]. The toxicity of intermediate lysozyme species, as well as that of the fibrils, have been investigated using cell-based systems, in which lysozyme oligomers and fibrils have been found to induce cell death via different mechanisms [12,13]. Previously, we published the results of a study using a *Drosophila melanogaster* model of lysozyme amyloidosis, where expression of the disease-associated variant F57I in the retina of the fly led to a disrupted eye phenotype, degradation of the unstable lysozyme protein and up-regulation of the unfolded protein response [14]. Using this fly model, we also investigated the effects of co-expressing F57I with SAP in the central nervous system (CNS) of the flies [15]. In the latter study, we found that SAP can overcome the toxic effect caused by expressing F57I in the fly CNS; the data suggested that SAP counteracts the formation of toxic F57I species in the flies. In the present study, double transgenic lysozyme flies were generated and the impact of SAP on aggregated lysozyme species was characterised in detail using histochemical assays and spectral analyses, where the fly brain was stained with two different anti-lysozyme antibodies, ab36362 and ab108508, and by the amyloid binding luminescent conjugated oligothiophenes (LCOs), h-FTAA and p-FTAA. LCOs are fluorescent molecules with flexible thiophene backbones that allow them to bind to different protein structures, thus giving rise to specific spectral properties depending on the structure with which they are interacting [16–20]. We found that SAP can counteract the toxicity induced by the expression of F57I by promoting the formation of alternative morphologies of aggregated lysozyme with more amyloidogenic characteristics. We tested the effect of SAP on *in vitro* lysozyme fibril formation, using the well-studied I59T lysozyme system [21,22] and confirmed that the presence of SAP promoted enhanced ThT fluorescence of the fibrils formed. Our findings suggest that a general mechanism for SAP's role in amyloid diseases may be to promote the formation of stable, amyloid-like fibrils, thereby decreasing the impact of toxic non-amyloidogenic species formed during the aggregation process.

## Materials and methods

### *Drosophila* stocks

To direct protein expression to the fly CNS, the GAL4/UAS system [23] with the post mitotic nsyb-Gal4 as the driver line [24], was used. Transgenic fly lines carrying genes encoding WT and F57I lysozyme were previously generated [14] and transgenic flies carrying the SAP gene were kindly provided by E. Lundgren. Flies with two copies of the lysozyme gene were generated by crossing *UAS-lysozyme/Cyo;nSyb-gal4/TM6B* flies with *UAS-lysozyme/UAS-lysozyme;+/+* flies. Flies with two copies of the lysozyme gene and the gene encoding SAP were generated by crossing *UAS-lysozyme/Cyo;nSyb-gal4/TM6B* flies with *UAS-lysozyme/Cyo;SAP/TM6B*. Crosses for longevity and staining assays were set up at 25°C, 60% RH. After the flies hatched, experiments were carried out at 29°C, 60% RH with a 12:12h light:dark cycle using female offspring. For staining assays, flies were aged for 25 days at 29°C and the heads were embedded in Tissue-Tek OCT compound.

### Longevity assay

The longevity assay was carried out as previously described [15], however the set-up temperature for the crosses was lowered to 25°C and the longevity assay was carried out at 29°C after the flies eclosed.

### Antibody staining

Staining of *Drosophila* brain sections was carried out after ageing the flies for 25 days following eclosion. Sections (10 μm) were taken from the OCT blocks using a Microm HM 550 Cryostat (Microm International GmbH), then they were placed on Superfrost Plus slides (Menzel Gläser) and stored at -20°C until required for use. The sections were fixed in 4% w/v PFA for 10 min at room temperature (RT) and the slides were washed in PBS (3x 3min) followed by permeabilisation using 0.5% Tween-20 (in PBS) for 15 min at RT. This was followed by additional washing steps (PBS, 3x 3 min). The rest of the staining protocol was carried out as previously described in [15] when using ab36362 and ab45151 (Abcam). For staining with ab108508 and ab27313 (Abcam), the same protocol as described above was used and both antibodies were diluted 1:500.

### LCO staining

Staining of *Drosophila* brain sections using p-FTAA, after ageing the flies for 25 days (double-expressing flies) or 35 days (single-expressing flies) following eclosion, was carried out as previously described [25]. For h-FTAA staining, a 1.5 mM stock solution of the probe was diluted 1:500 using PBS, and staining was carried out in the same manner as described for p-FTAA.

### Emission spectra recording and fluorescence lifetime imaging (FLIM)

A Zeiss LSM 780 confocal microscope was used for fluorescent imaging; the images were processed using LSM software or Adobe Photoshop. All images were treated identically. To detect lysozyme species by emission spectroscopy, samples were excited with a laser tuned to 488 nm and emission spectra were recorded using a tunable In Tune laser (488–640 nm). Spectra were collected from a minimum of five individual spots within a minimum of three different fly brains. Fluorescence life time imaging was carried out as described in [18], using 63x magnification.

### Statistics

To analyse the longevity data, Kaplan-Meier survival curves were generated using Graph Pad Prism and log rank statistics were calculated.

### I59T *in vitro* fibril formation in the absence and presence of SAP

I59T human lysozyme was expressed and purified as previously described [21] and Serum amyloid P (SAP) component was purchased from Sigma-Aldrich (UK) Ltd. (Gillingham, UK). Prior to using the SAP, the stock solution was buffer exchanged using a Zeba spin desalting column (7 kDa cut-off; Thermo Fisher Scientific, Hemel Hempstead, UK) into 0.1M sodium citrate buffer (pH 5.0). Aggregation studies were performed with I59T lysozyme (3.4 μM, 0.1 M sodium citrate buffer pH 5.0) in the absence or presence of SAP (3.4 μM) in 1 cm quartz cuvettes. Samples were incubated with stirring at 60°C in a Cary Eclipse spectrofluorimeter (Agilent Technologies (UK) Ltd., Cheadle, UK). Light scattering was monitored at 500 nm with slit widths of 5 nm. SAP alone (3.4 μM) was also incubated under aggregation conditions. Experiments were performed in triplicate. Aliquots (20 μL) were removed from the samples at different time points (0, 140, 285, 360 and 1400 min) and immediate flash frozen in liquid nitrogen until ThT analysis.

### Thioflavin-T analysis

In a total volume of 100 μL, 10 uL sample was mixed with 10 uL ThT (1.5 mM) in 0.1 M citrate buffer (pH 5.0). The ThT fluorescence was monitored in duplicate per sample as measured using bottom-optics in a Fluorstar Omega plate reader (BMG Labtech, Aylesbury, UK) with 440 nm and 480 nm excitation and emission filters, respectively. Endpoint ThT emission scans were measured in a Cary Eclipse spectrofluorimeter with excitation at 440 nm (5 nm slit width) and emission spectra recorded between 475–600 nm. Samples contained 40 μL endpoint aggregation sample, 40 μL ThT (1.5 mM stock) and 320 μL 0.1 M citrate buffer (pH 5.0) in a 1 cm quartz cuvette.

### Transmission Electron Microscopy (TEM)

Samples for TEM were prepared on carbon support film, 400 mesh, 3 mm copper grids (EM Resolutions Ltd., Sheffield, UK) and stained with 2% (w/v) uranyl acetate. The samples were imaged on a FEI Tecnai G2 transmission electron microscope in the Cambridge Advanced Imaging Centre (CAIC, University of Cambridge, Cambridge, UK). Images were analysed using the SIS Megaview II Image Capture system (Olympus, Tokyo, Japan).

## Results

### LCO staining indicates that F57I lysozyme aggregates have greater amyloid-like properties in the presence of SAP in *Drosophila* CNS

**Fig 1:**
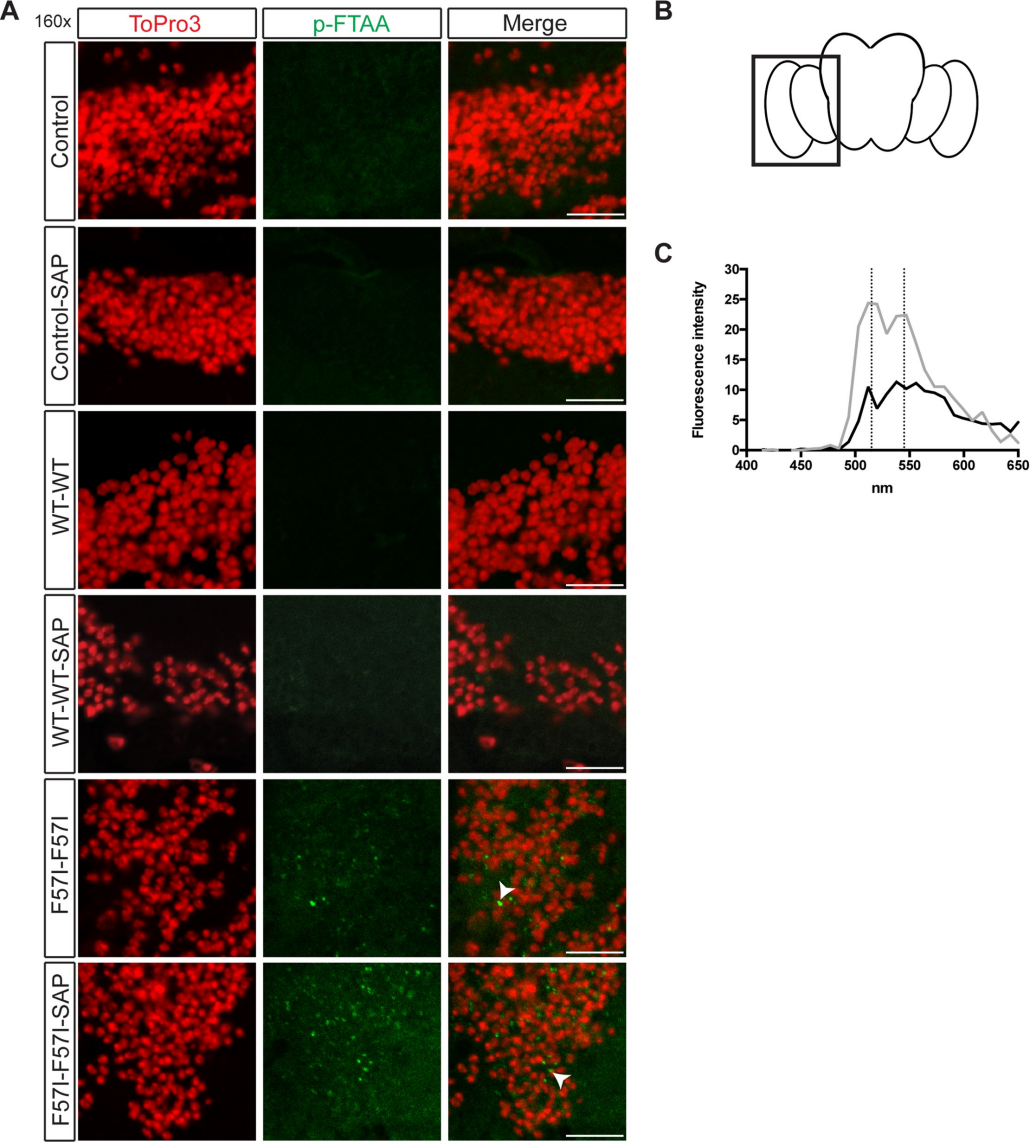
Double-peak emission spectra of p-FTAA in F57I-F57I-SAP flies indicates the formation of alternative aggregated lysozyme morphologies in these flies. (A) *Drosophila* brain sections (day 25) stained with the amyloidogenic probe p-FTAA (green) and counter-stained with ToPro3 (red) to visualise cell nuclei. A p-FTAA positive signal was detected only in the F57I-F57I and F57I-F57I-SAP flies (indicated with white arrow heads in the merged images). (B) A schematic fly brain, indicating the area of the brain shown in the micrographs. (C) When recording the emission spectra for the signal detected using p-FTAA, the characteristic double peak emission spectra, with peaks at 515 and 545 nm, was not observed for the F57I-F57I flies (black), whereas it was detected in the F57I-F57I-SAP flies (grey). Micrographs show 160x magnification, scale bar = 10 μm, n = 9 for p-FTAA staining and recorded emission spectra.

The accumulation of large quantities of amyloid aggregates is characteristic of lysozyme amyloidosis. To investigate the formation of amyloid aggregates in our lysozyme flies, flies expressing F57I or WT lysozyme, with and without the co-expression of SAP, were stained using the amyloid binding conjugated luminescent oligothiophene (LCO) p-FTAA. A variety of different amyloid fibrils have been probed *in vitro* using p-FTAA, e.g. lysozyme, insulin and the prion protein [16], and p-FTAA has also been shown to detect both *in vitro* Aβ fibrils and amyloid aggregates within the brain tissue of transgenic AD mice and *Drosophila* [25–27]. Upon binding to amyloidogenic structures, p-FTAA gives rise to characteristic emission spectra, with double peaks found at 515 and 545 nm [17,26]. Initially, the gene expression was directed to the fly CNS using the post mitotic driver, nsyb-Gal4, however, no amyloid aggregates were detected by p-FTAA in F57I or WT flies, with and without SAP, when aged for 35 days (S1 Fig). Next, lysozyme flies with two copies of the lysozyme gene were generated to increase the expressed lysozyme levels, and brain sections of *Drosophila*, aged for 25 days, were stained with p-FTAA. A perinuclear positive p-FTAA signal was detected in F57I-F57I and F57I-F57I-SAP flies (Fig 1A). No p-FTAA signal was observed in nsyb-Gal4 background *w*^*1118*^ control flies ± SAP (which do not express human lysozyme) or in WT-WT flies ± SAP (Fig 1A). The p-FTAA positive aggregates in the F57I-F57I ± SAP flies were located in the medulla of the fly brain (indicated in schematic fly brain shown in Fig 1B). The p-FTAA positive signal found in F57I-F57I-SAP had a double peak emission spectra, in which the peaks were located at 515 and 545 nm, characteristic of amyloidogenic aggregates (Fig 1C), while the emission spectra for the p-FTAA positive signal in F57I-F57I displayed only a single, slightly red-shifted emission peak (Fig 1C). Thus, when bound to aggregates present in the F57I-F57I-SAP flies, p-FTAA showed a spectroscopic signature similar to those obtained from other amyloid deposits, whereas the aggregates in F57I-F57I did not. Similar spectra to the F57I-F57I aggregates have previously been observed for p-FTAA bound to non-ThT positive, pre-fibrillar species of lysozyme [16].

**Fig 2:**
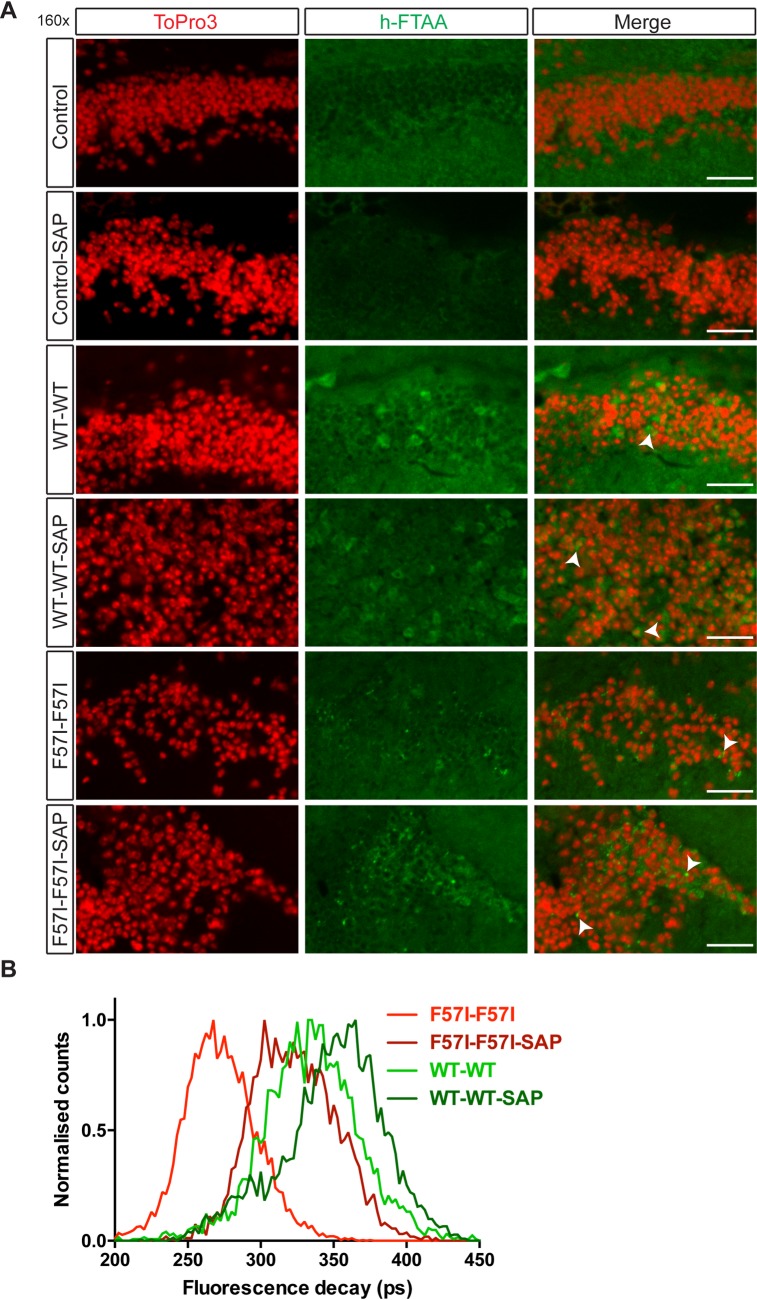
Co-expression of SAP changes the fluorescence lifetime distribution of h-FTAA in all lysozyme expressing flies. (A) *Drosophila* brain sections (day 25) stained with the amyloidogenic probe h-FTAA (green) and counter-stained with ToPro3 (red) to visualise the cell nuclei. h-FTAA positive signal was observed in the WT-WT and the F57I-F57I flies, with and without SAP (indicated with white arrow heads in the merged images). (B) Comparison of the fluorescence lifetime of h-FTAA in WT-WT and F57I-F57I, with and without SAP. An increase in fluorescence lifetime was observed in F57I-F57I-SAP and WT-WT-SAP flies compared to flies not expressing SAP. A clear difference in h-FTAA fluorescence lifetime was also observed between WT-WT and F57I-F57I flies. Micrographs show 160x magnification, scale bar = 10 μm, n = 8 for h-FTAA staining and n = 5 for fluorescence lifetime analysis.

To further characterise the aggregated species in our lysozyme flies, a second LCO, h-FTAA was used [28]. h-FTAA has previously been used to detect amyloid aggregates in tissue samples from patients suffering from systemic amyloidosis [19], as well as a variety of other disease-associated protein aggregates [18,29]. No h-FTAA positive signal was detected in control or control-SAP flies (Fig 2A). In both WT-WT and F57I-F57I flies, with and without SAP, perinuclear signals from h-FTAA were detected (Fig 2A). The location of these h-FTAA positive aggregates corresponded closely to the p-FTAA signal detected in the medulla. In previous studies, h-FTAA bound to amyloid structures gave rise to characteristic double-peak emission spectra [18,29], however, this was not observed for the h-FTAA signal detected in any of the lysozyme fly genotypes (S2 Fig). To further investigate the differential states of binding and to observe minute variations in h-FTAA-aggregate interactions, fluorescence lifetime imaging (FLIM) was used. Different forms of h-FTAA positive protein aggregates associated with distinct prion strains display variations in the fluorescence decay of h-FTAA [18]. There was a clear difference in the fluorescence lifetime distribution of h-FTAA when F57I-F57I and F57I-F57I-SAP flies were compared; the fluorescence decay measured in F57I-F57I flies (200–350 ps) was considerably lower than in F57I-F57I-SAP flies (250–400 ps) (Fig 2B). A similar trend was also seen for WT-WT flies, in which flies co-expressing SAP gave rise to increased fluorescence decays (Fig 2B). Interestingly, a clear difference in the distribution of h-FTAA lifetime decays between aggregates formed in F57I-F57I and WT-WT flies (200–350 and 250–425 ps respectively) was also observed. Taken together, the FLIM experiments showed that h-FTAA binds in different modes to the aggregates formed in the WT-WT and in F57I-F57I flies, both in the absence and in the presence of SAP.

### Antibody staining reveals co-localisation with SAP and lysozyme at different locations in the fly brain

**Fig 3:**
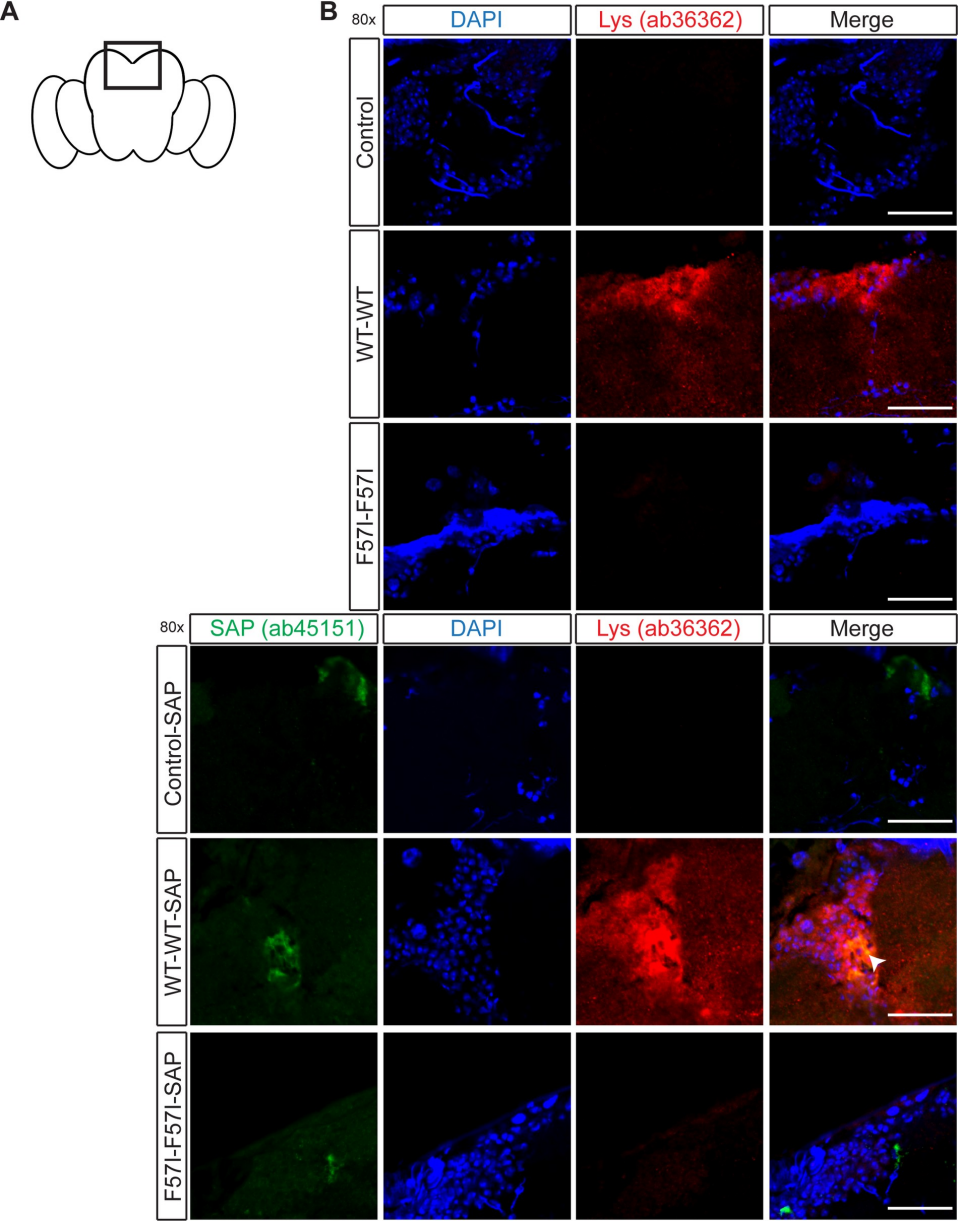
Detection of lysozyme and co-localisation with SAP in the superior medial protocerebrum of the WT-WT fly brain. (A) A schematic fly brain, indicating the areas of the brain shown in the micrographs. (B) *Drosophila* brain sections (day 25) stained with an anti-lysozyme antibody (ab36362, red) or co-stained with anti-lysozyme antibody (ab36362, red) and an anti-SAP antibody (ab45151, green), then counter-stained with DAPI (blue) to visualise the cell nuclei. Co-localisation between lysozyme and SAP is observed as yellow areas (indicated by the white arrow in the merged images). Micrographs show 80x magnification, scale bar = 25 μm, n = 5.

Next, we probed the location of lysozyme in the fly CNS and investigated whether lysozyme and SAP co-localised using two different anti-lysozyme antibodies and an anti-SAP antibody. Sections of *Drosophila* brains were collected on day 25 and initially stained using the anti-lysozyme antibody ab36362, a mouse monoclonal antibody raised against full-length native human lysozyme, and the anti-SAP antibody ab45151. The sections were counterstained with DAPI to visualise the cell nuclei (Fig 3B). In all SAP expressing flies, a signal from SAP was detected, and a lysozyme signal was observed in WT-WT flies while no lysozyme was detected in the F57I-F57I flies (Fig 3B). Lysozyme and SAP were observed to be co-localised in the WT-WT-SAP flies whereas no lysozyme signal was observed in the F57I-F57I-SAP flies. The majority of the lysozyme detected in WT-WT flies, with or without the presence of SAP, was found to be located in the superior medial protocerebrum (SMP), as indicated on the schematic image of a fly brain in Fig 3A. Co-localisation with SAP was most pronounced in this area of the brain.

**Fig 4:**
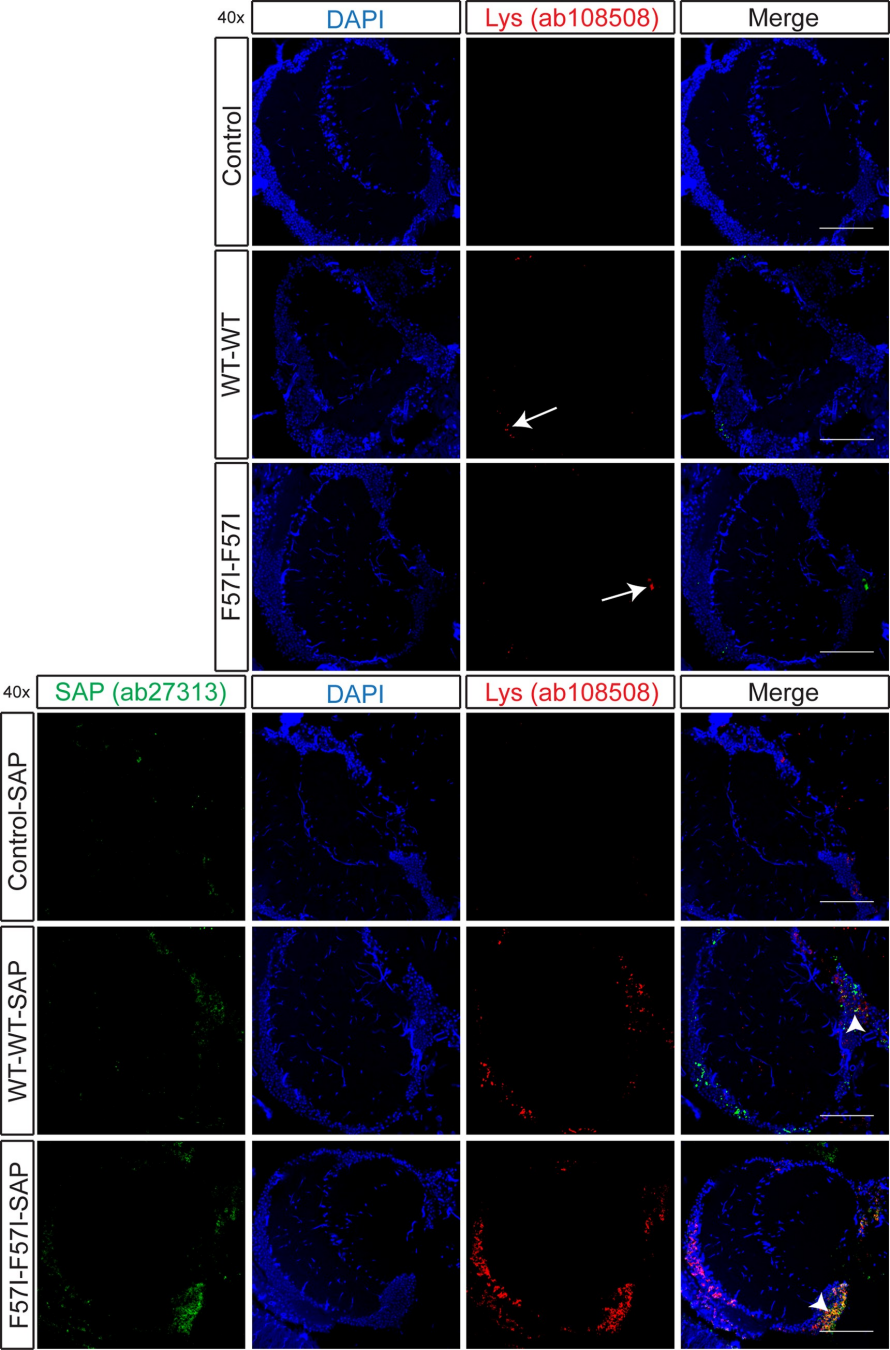
Detection of lysozyme and co-localisation with SAP in the medulla of the WT-WT and F57I-F57I fly brain. *Drosophila* brain sections (day 25) stained with an anti-lysozyme antibody (ab108508, red) or co-stained with anti-lysozyme antibody (ab108508, red) (highlighted with white arrows in the lysozyme panel for WT-WT and F57I-F57I flies) and an anti-SAP antibody (ab27313, green), then counter-stained with DAPI (blue) to visualise the cell nuclei. Co-localisation between lysozyme and SAP is observed as yellow areas (indicated by the white arrow heads in the merged images). Micrographs were taken at 40x magnification, scale bar = 50 μm, n = 5.

To further investigate the presence of lysozyme in *Drosophila* brain sections, an additional lysozyme antibody was used, ab108508, a rabbit monoclonal antibody raised using a synthetic peptide corresponding to amino acids 50–70 of human lysozyme. Using this antibody, a weak lysozyme signal was observed in both WT-WT and F57I-F57I flies (Fig 4). Interestingly, the signal from ab108508 was found mainly in the medulla, the same area where the p- and h-FTAA positive species were detected (as indicated on the schematic fly brain in Fig 1A), rather than in the SMP where ab36362 lysozyme detection was observed only in the WT-WT flies. When staining for lysozyme in the flies co-expressing lysozyme and SAP, an increase in the lysozyme signal was detected in both WT-WT-SAP flies and F57I-F57I-SAP flies (Fig 4). To investigate co-localisation between lysozyme and SAP, the anti-lysozyme antibody, ab108508, was used together with a new anti-SAP antibody (ab27313). A SAP signal was detected in all SAP expressing flies, and in both the WT-WT-SAP and F57I-F57I-SAP flies, co-localisation between lysozyme and SAP was detected (Fig 4), with the signal appearing to be stronger for the F57I-F57I-SAP flies. In summary, lysozyme was detected in different locations of the fly brain using two different lysozyme antibodies. The antibody, ab108508, was able to detect lysozyme in WT-WT and F57I-F57I flies, with and without SAP in the same area of the fly brain where the p- and h-FTAA positive species were detected indicating that these p- and h-FTAA positive species are composed of lysozyme. The antibody ab36362 detected lysozyme only in the WT-WT flies ± SAP.

### Co-expression of SAP reduces the toxicity observed in a longevity assay of the F57I-F57I flies

**Fig 5:**
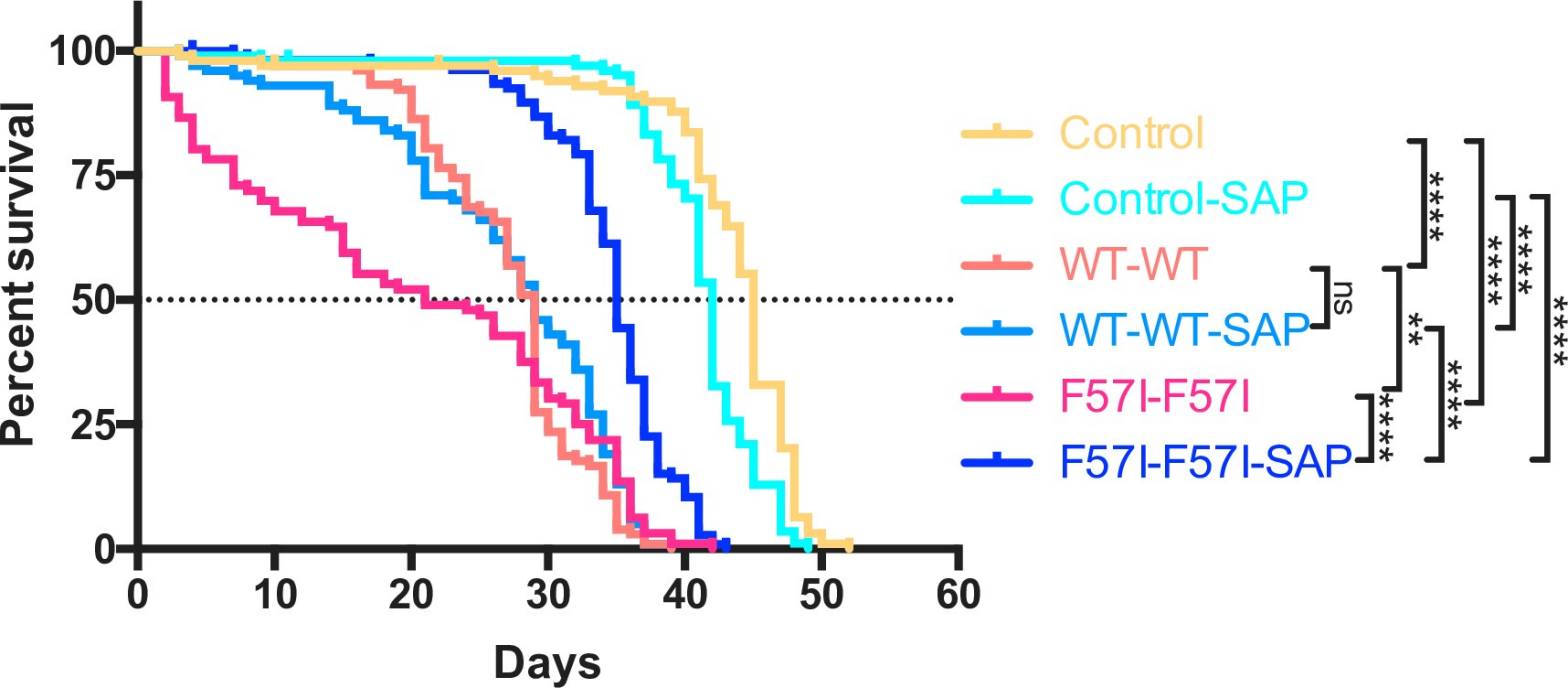
SAP ameliorates the reduction in median survival time of the F57I-F57I flies. Survival trajectories for control, WT-WT and F57I-F57I flies, with and without co-expression of SAP. WT-WT and F57I-F57I flies, with and without SAP, displayed significant decreases in their median survival times compared to control flies (p < 0.0001). Co-expression of SAP increased the median survival time for the F57I-F57I flies by 14 days (35 and 21 days, respectively) (p < 0.0001), whereas co-expression of SAP in WT-WT flies had no effect on the median survival time (29 days for both genotypes). Kaplan-Meier graph showing the percentage of survival, n = 100.

To investigate whether co-expression of SAP impacts the median survival times of lysozyme flies containing two copies of the lysozyme gene, a longevity assay was carried out. The median survival times of control flies and of control-SAP flies were 45 and 42 days, respectively and the median survival time of WT-WT flies and of F57I-F57I flies were 29 and 21 days, respectively (Fig 5). Both WT-WT and F57I-F57I flies showed a significant decrease in their median survival times compared to control flies (p < 0.0001). The median survival time of F57I-F57I flies was significantly shorter compared to WT-WT flies (p < 0.01), revealing that there was a toxic effect in both the WT-WT and F57I-F57I flies that was stronger for the F57I-F57I flies as compared to the WT-WT flies (Fig 5). The corresponding survival times with co-expression of SAP (WT-WT-SAP and F57I-F57I-SAP flies) were 29 and 35 days respectively (Fig 5). Thus, no significant rescue by SAP was found in the WT-WT flies whereas co-expression of SAP had a significant rescue effect in the F57I-F57I flies (p < 0.0001), in which the median survival time was increased by 14 days (from 21 to 35 days) resulting in a significantly higher medium survival time of the F57I-F57I-SAP flies compared to the WT-WT-SAP flies (p < 0.0001) (Fig 5). The longevity assay showed that the toxicity, observed as a decrease in median survival time, was induced by expressing two copies of the F57I gene in *Drosophila* CNS, and could be countered by co-expressing SAP. Interestingly, SAP has no effect on the toxicity induced by expressing two copies of the WT lysozyme gene.

### Dual effect of SAP on *in vitro* lysozyme fibril formation

**Fig 6:**
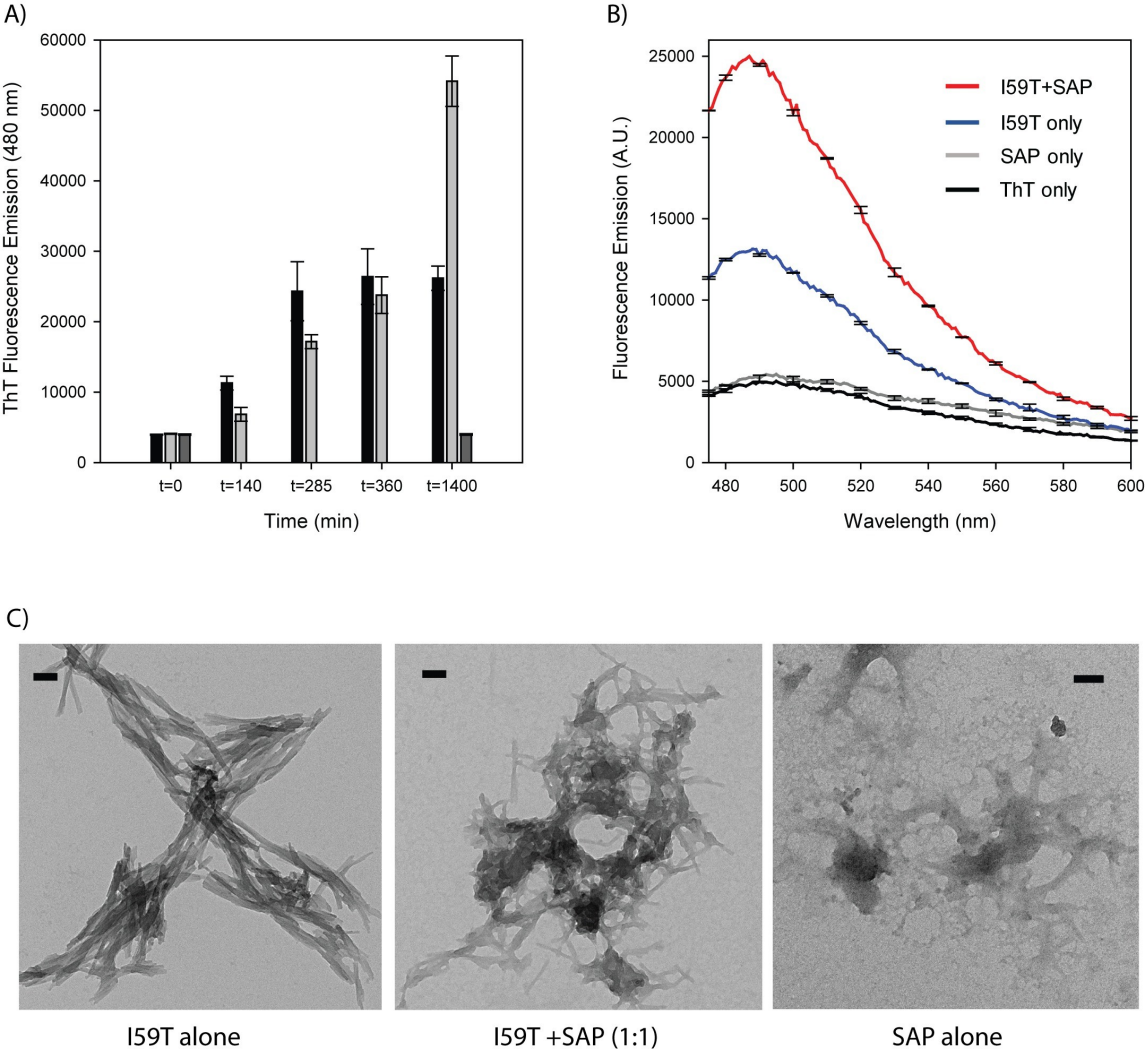
The effect of SAP on *in vitro* fibril formation of I59T lysozyme. I59T lysozyme (3.4 μM) alone (black bars), I59T with SAP (1:1 molar ratio; grey bars) and SAP alone (3.4 μM; dark grey bars) were incubated at 60°C with stirring and time points were analysed for ThT fluorescence emission (480 nm), in duplicate (A). ThT emission spectra for ThT alone (black), SAP alone (grey), I59T alone (blue) and I59T with SAP (red), analysed in triplicate (B). Representative TEM images for the aggregation samples (C). Scale bar is 100 nm.

Given the relatively high native-state stability of human lysozyme, including the disease-related variants, *in vitro* fibril formation conditions often rely on extreme solution conditions such as low pH, use of denaturants, and/or high NaCl content [30,31]. Using an non-natural variant of human lysozyme, I59T, it is possible to study the process at pH 5, 60°C in the absence of denaturants or NaCl and at low protein concentrations (2–10 μM) [21]. This variant has been used to explore the inhibition activity of a number of extracellular chaperones (clusterin, haptoglobin and A2M) [32,33] and therefore, we tested the effects of SAP on I59T fibril formation. When I59T was incubated with a 1:1 molar ratio (lysozyme-to-monomeric SAP), we found the effect on the process of fibril formation to be relatively subtle; the I59T with SAP appeared to have a slight delay in fibril formation (at t = 140 min and t = 285 min, the ThT signal was lower for I59T+SAP than for I59T alone), but by 1400 min, both samples had fully aggregated (Fig 6A). Interestingly, when the endpoint fibril species for I59T and I59T+SAP were analysed by ThT fluorescence emission, we saw that the fibrils formed in the presence of SAP gave enhanced ThT fluorescence relative to the I59T fibrils alone (Fig 6B). The SAP alone, incubated under the same conditions, formed aggregates but these were ThT negative. The morphology of all the aggregates were confirmed by TEM, with the I59T and the I59T+SAP giving typical fibrillar morphologies and the SAP showing amorphous type aggregates (Fig 6C).

## Discussion

What role SAP plays in amyloid diseases is still a subject of debate. This study aimed to achieve a better understanding of how SAP prevents apoptosis and restores the lifespan of F57I lysozyme expressing flies [15] and to examine the effect of SAP on the aggregation process of lysozyme.

### SAP co-localises with lysozyme aggregates in the fly medulla

The result from the LCO staining of the *Drosophila* brain sections showed that both p- and h-FTAA detected aggregated species in F57I-F57I ± SAP, whereas in the WT-WT ± SAP flies, only h-FTAA positive aggregates were detected. Using the anti-lysozyme antibody, ab108508, revealed that lysozyme species in both F57I-F57I ± SAP and WT-WT ± SAP flies are located in the medulla of the fly brains, strongly suggesting that ab108508 and the LCOs can detect the same species: insoluble/aggregated forms of lysozyme. SAP was found to co-localise with these species.

### SAP reduces toxicity in F57I-F57I flies but not in WT-WT flies

In the longevity experiments, crosses were set up at 25°C because at 29°C, no adult flies developed from the pupae stage for the F57I-F57I flies, revealing that expression of two copies of the F57I gene in the fly CNS is lethal during metamorphosis at 29°C. Data from the longevity assay revealed a toxic effect in both F57I-F57I and WT-WT flies. However, while SAP was able to extend the lifespan of the F57I-F57I flies, co-expression of SAP resulted in no change in the lifespan of the WT-WT flies. This suggests clear differences in the mechanisms of toxicity between the F57I-F57I and WT-WT flies.

### SAP promotes the formation of amyloid-like lysozyme aggregates in F57I-F57I flies

The spectral properties of the LCOs differ depending on the structure to which they are bound, as well as the environment surrounding them. When the fluorescence decay of h-FTAA was analysed, SAP increased the fluorescence decay lifetime for F57I lysozyme aggregates. This was also the case for WT lysozyme aggregates but to a lower extent. Changes in the fluorescence decay lifetime indicate that co-expression with SAP results in structural alterations of the lysozyme species detected by h-FTAA. Fluorescence decay also differed between h-FTAA positive species in F57I-F57I and WT-WT flies, indicating that the aggregation process of the two lysozyme variants results in structurally different h-FTAA positive aggregates. FLIM analysis of two different mouse models for prion disease has been performed using h-FTAA, and this was found to give rise to distinct fluorescence decay lifetimes depending on the prion strain associated with the aggregates [18]. Interestingly, a shorter fluorescence decay was observed for the h-FTAA positive prion aggregates in the sheep scrapie mice compared with the fluorescence decay observed for aggregates detected in a mouse model for chronic wasting disease (CWD). The terminal onset for scrapie mice occurs much earlier than for CWD mice [34], indicating that there is a greater toxic effect in the scrapie mice, in which h-FTAA fluorescence decay was observed to be shorter compared to that observed in the CWD mice [18]. Since the shortest fluorescence decay was observed for the lysozyme species detected in the F57I-F57I flies, these species may possess toxic properties and thus be responsible for the reduced life span of the F57I-F57I flies.

For the other LCO, p-FTAA, binding to mature amyloid aggregates gives rise to a double peak emission spectra, which was observed for the p-FTAA positive species in F57I-F57I-SAP flies, but not in F57I-F57I flies. This indicates that co-expression of SAP changes the structure of the p-FTAA positive species, promoting the formation of aggregates that possess more amyloid-like characteristics. This is supported by our *in vitro* fibril formation studies, where the endpoint I59T+SAP fibrils have enhanced ThT fluorescence compared to the I59T fibrils alone. This enhanced ThT fluorescence of I59T lysozyme fibrils in the presence of SAP may suggest that SAP is able to alter the overall β-sheet arrangement within the I59T lysozyme fibrils. Alternatively, SAP may interact with the fibrils to change their structural orientation relative to the lysozyme fibrils alone, however, our TEM images did not reveal significant differences in the morphologies of the I59T fibrils, in the absence or presence of SAP.

Interestingly, in our *in vitro* analysis, SAP appeared to delay fibril formation of I59T within the first hours of the aggregation process. A similar phenomenon was detected *in vitro* by Ozawa et al. where SAP, in the presence of Ca^2+^, was found to first inhibit amyloid fibril formation of D76N β2-microglobulin (β2-m) and then accelerated D76N β2-m amyloid fibril formation [35]. The study showed that doughnut-shaped pentameric SAP coated the surface of the D76N β2-m fibrils. The authors suggested the possibility that SAP can exhibit both anti-amyloidogenic activity, by interacting with soluble oligomers and unfolded momomers via the A face and pro-amyloidogenic activity, by binding to the surface of amyloid fibrils via the B face. Our *in vitro* data for I59T fibril formation in the presence of SAP clearly support this model and strengthens the suggestion that the anti- and pro-amyloidogenic activities of SAP can be present simultaneously.

In summary, the presence of ab108508-, h-FTAA- and p-FTAA positive lysozyme species in the F57I-F57I flies results in a toxic effect that can be rescued through interactions between these species and SAP. This interaction promotes the formation of alternative structures with increased amyloid-like properties, thereby reducing the number of toxic species in the F57I-F57I flies. The toxicity detected in WT-WT flies cannot be rescued by SAP and the aggregated species detected by h-FTAA and ab108508 do not differ substantially in their spectral properties with and without SAP. Thus, it is possible that the toxic effect detected in the WT-WT flies is caused by the ab108508 and h-FTAA positive lysozyme species that are formed in these flies, and since SAP does not affect their structure, the toxicity remains even in the presence of SAP. An alternative explanation for the toxicity in the WT-WT flies may be due to the presence of high protein levels of WT lysozyme, as confirmed by ab36362 antibody detection, showing the presence of protein throughout the entire brain of the WT-WT ± SAP flies. Expression of WT lysozyme is toxic during metamorphosis when it is expressed ubiquitously [14], likely due to its enzymatic activity.

The question of whether smaller, non-amyloidogenic species or mature amyloid aggregates are the main toxic species in amyloid disease has been extensively investigated during recent years; however, evidence suggests that both possess toxic properties. In our *Drosophila* model of lysozyme amyloidosis, the toxic effect observed appears to arise from intermediate species of F57I formed on the pathway leading to the more amyloidogenic-like lysozyme aggregates observed in the presence of SAP. Toxicity studies have shown that larger oligomeric lysozyme species induce toxicity in different cell cultures [36], and both oligomers and fibrils have been observed to cause cell death, albeit via different mechanisms [13]. Less structured lysozyme aggregates, i.e. aggregates with a larger non-core region, have been found to induce a higher level of toxicity compared to more structured aggregates [30]. The evidence reported for the role of SAP in amyloid disease is also contradictory; SAP has been found to both accelerate and suppress aggregation of the Aβ peptide and to bind to and stabilise amyloid aggregates [1,37–39].

Overall, we suggest that the toxic effect detected in the F57I-F57I flies is caused by the ab108508-, h-FTAA- and p-FTAA positive species and that SAP can bind to these species and promote the formation of less toxic amyloid-characteristic structures. Thus, if the pathological event in lysozyme amyloidosis is due to toxicity caused by intermediate lysozyme aggregates, an increased presence of SAP could potentially rescue toxicity by reducing the pool of these toxic species. On the other hand, if the toxicity in lysozyme amyloidosis is due to the massive accumulation of amyloid aggregates in internal organs, the presence of SAP could potentially promote the formation of these amyloid species and thereby worsen the disease progression. Clearly, the question whether SAP possesses a beneficial or an adverse effect in lysozyme amyloidosis and in other amyloid diseases require further investigations.

## Supporting information

S1 Fig p-FTAA staining of single-lysozyme expressing flies. *Drosophila* brain sections of control, WT and F57I flies, with and without SAP, aged for 35 days and stained with the LCO p-FTAA and cell nuclei (red) visualized using ToPro3. No p-FTAA positive species were observed in either fly genotype. Micrographs were taken at 40x magnification, scale bar = 50 μm, n = 5.
(PDF)

S2 Fig h-FTAA emission spectra. Recorded emission spectra for (A) WT-WT, (B) WT-WT-SAP, (C) F57I-F57I and (D) F57I-F57I-SAP when stained with the LCO h-FTAA. No double-peak emission spectra were detected, n = 9.
(PDF)

S1 Data (XLSX)

## Abbreviations

CNS: central nervous system
F57I: disease-associated lysozyme variant
FLIM: fluorescent lifetime imaging
h-FTAA: heptameric formic thiophene acetic acid
LCOs: luminescent conjugated oligothiophenes
p-FTAA: pentameric formic thiophene acetic acid
SAP: serum amyloid P component
ThT: thioflavin-T
WT: wild type
