# Supplementary figures and images for: Serum amyloid P component promotes formation of distinct aggregated lysozyme morphologies and reduces toxicity in Drosophila flies expressing F57I lysozyme
Source: PLoS One. 2020 Jan 24;15(1):e0227227. doi: 10.1371/journal.pone.0227227 (PMC6980568; doi:10.1371/journal.pone.0227227)

40x

Control

WT

F57I

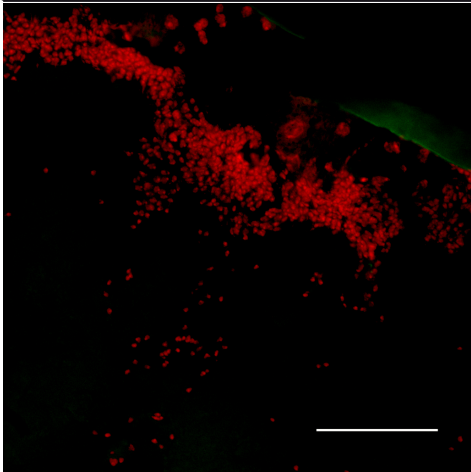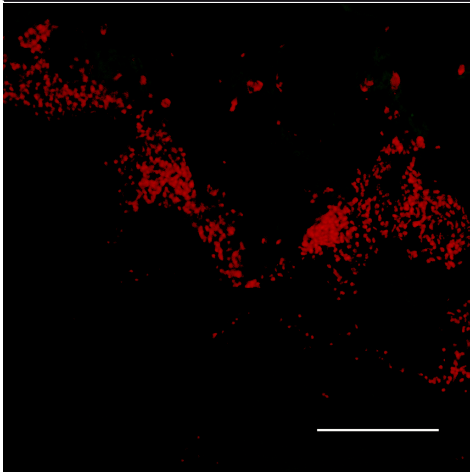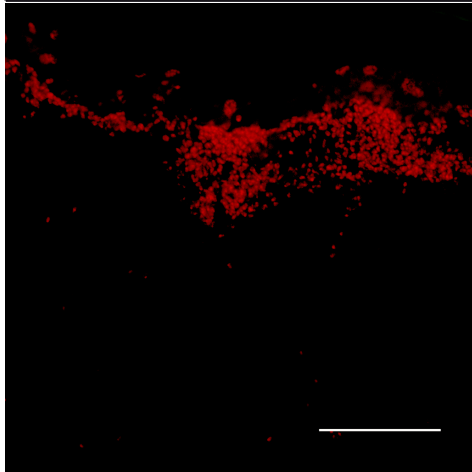

Control-SAP

WT-SAP

F57I-SAP

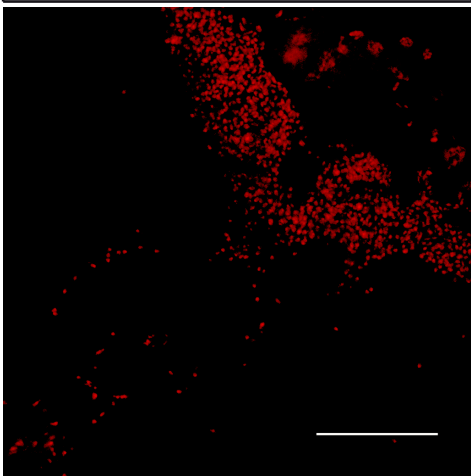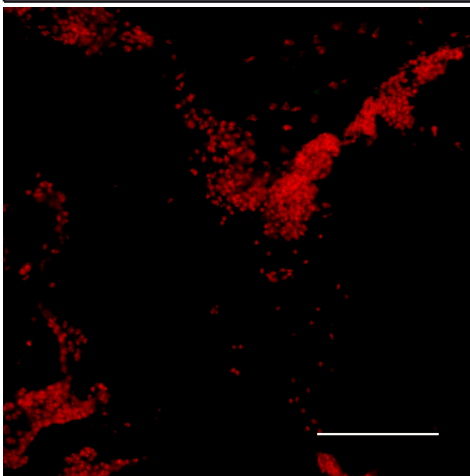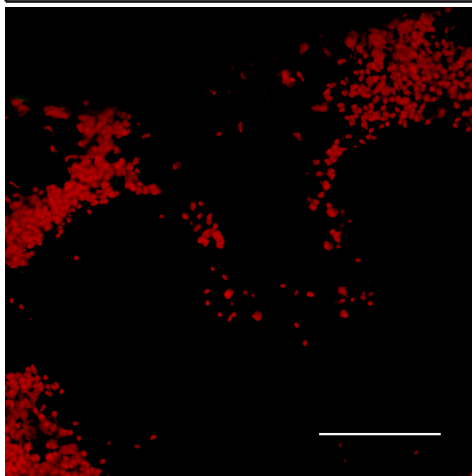

Supplement: S1 Fig — Drosophila brain sections of control, WT and F57I flies, with and without SAP, aged for 35 days and stained with the LCO p-FTAA and cell nuclei (red) visualized using ToPro3. No p-FTAA positive species were observed in either fly genotype. Micrographs were taken at 40x magnification, scale bar = 50 μm, n = 5. (PDF) [file pone.0227227.s001.pdf]

**A**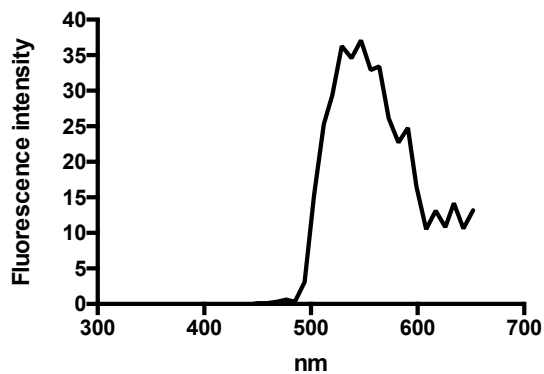**B**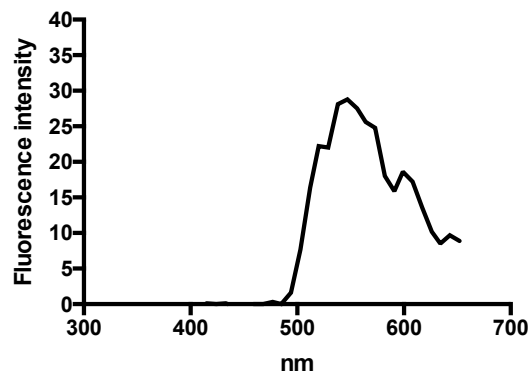**C**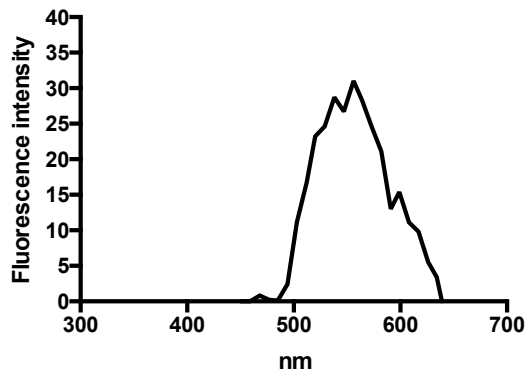**D**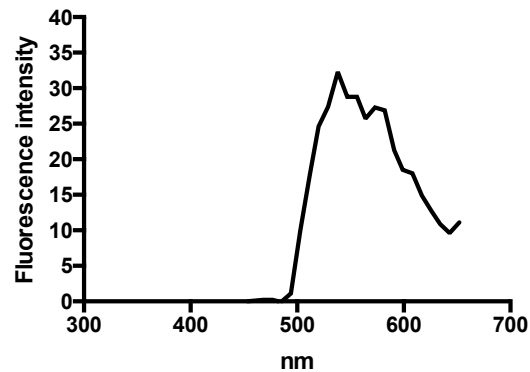

Supplement: S2 Fig — Recorded emission spectra for (A) WT-WT, (B) WT-WT-SAP, (C) F57I-F57I and (D) F57I-F57I-SAP when stained with the LCO h-FTAA. No double-peak emission spectra were detected, n = 9. (PDF) [file pone.0227227.s002.pdf]
